# A Society-to-Cells approach to evaluating multilevel and interrelated drivers of breast cancer disparities in Black women

**DOI:** 10.1038/s41523-025-00812-0

**Published:** 2025-08-13

**Authors:** Maurade Gormley, Wayne R. Lawrence, Jesse J. Plascak, Electra D. Paskett, Coral Omene, Adana A. M. Llanos

**Affiliations:** 1 School of Nursing, University of Connecticut, Storrs, CT USA; 2 Department of Epidemiology and Biostatistics, University at Albany, State University of New York, Rensselaer, NY USA; 3 Division of Cancer Prevention and Control, Department of Internal Medicine, College of Medicine, The Ohio State University, Columbus, OH USA; 4 Comprehensive Cancer Center, The Ohio State University, Columbus, OH USA; 5 Rutgers Cancer Institute, Rutgers University, New Brunswick, NJ USA; 6 Department of Medicine, Robert Wood Johnson Medical School, New Brunswick, NJ USA; 7 Department of Epidemiology, Mailman School of Public Health, Columbia University Irving Medical Center, New York, NY USA; 8 Herbert Irving Comprehensive Cancer Center, Columbia University Irving Medical Center, New York, NY USA

**Keywords:** Breast cancer, Cancer epidemiology

## Abstract

Despite advances in detection and treatment, Black women in the US continue to experience more aggressive breast cancer subtypes and higher mortality. Framed through a *Society-to-Cells* lens, this review presents a comprehensive framework for understanding how multilevel drivers—from structural forces to cellular responses—interact to perpetuate disparities. Addressing these inequities requires systemic reforms targeting root causes, including policies to redress historical neighborhood disinvestment and eliminate bias within healthcare systems.

## Introduction

Approximately 3.8 million breast cancer survivors currently reside in the United States (US)^1^. While advances in early detection and targeted therapies have significantly improved breast cancer survival overall, these gains have not been equitably realized by Black women, who continue to bear a disproportionate burden of breast cancer mortality^2–4^. Despite having slightly lower incidence rates and higher screening rate^5^, Black women experience a 38 to 41% higher mortality rate from breast cancer compared to non-Hispanic White (NHW) women^2,5,6^. Although racial disparities in cancer outcomes have narrowed across all cancers combined, they have widened specifically for breast cancer among Black women^2^. As such, breast cancer remains one of the most striking and inadequately explained disparities affecting Black women^7^. Differences in tumor biology^8–10^—such as higher prevalence of triple-negative subtype and epigenetic alterations—and inequities in care, including delayed diagnosis, delayed treatment initiation, and suboptimal treatment^11,12^ partially contribute to the mortality gap, but do not fully explain the persistent inequities in outcomes. Black women continue to experience worse breast cancer outcomes even after accounting for age, comorbidities, socioeconomic status (SES), and tumor clinicopathologic characteristics^13–15^. This enduring disparity, which cannot be fully attributed to individual-level clinical or sociodemographic factors alone, underscores the need for a comprehensive, multilevel framework that addresses upstream structural drivers. Structural racism, racialized residential segregation, and systemic disinvestment shape the social and physical environments in which Black women live, work, and receive care, influencing a range of downstream factors—including neighborhood stability, access to high-quality healthcare, and provider bias. These interrelated systems of structural disadvantage contribute not only to inequities in care delivery, but also to cumulative physiologic burden^16,17^. Chronic exposure to adverse social conditions activates stress response systems in ways that become biologically embedded over time, further exacerbating disparities in breast cancer outcomes^16,18^. These patterns underscore the fact that racial disparities in breast cancer mortality are not the result of individual behaviors, but are deeply rooted in structural and social inequities^16,19^.

## Conceptual framework

**Fig. 1:**
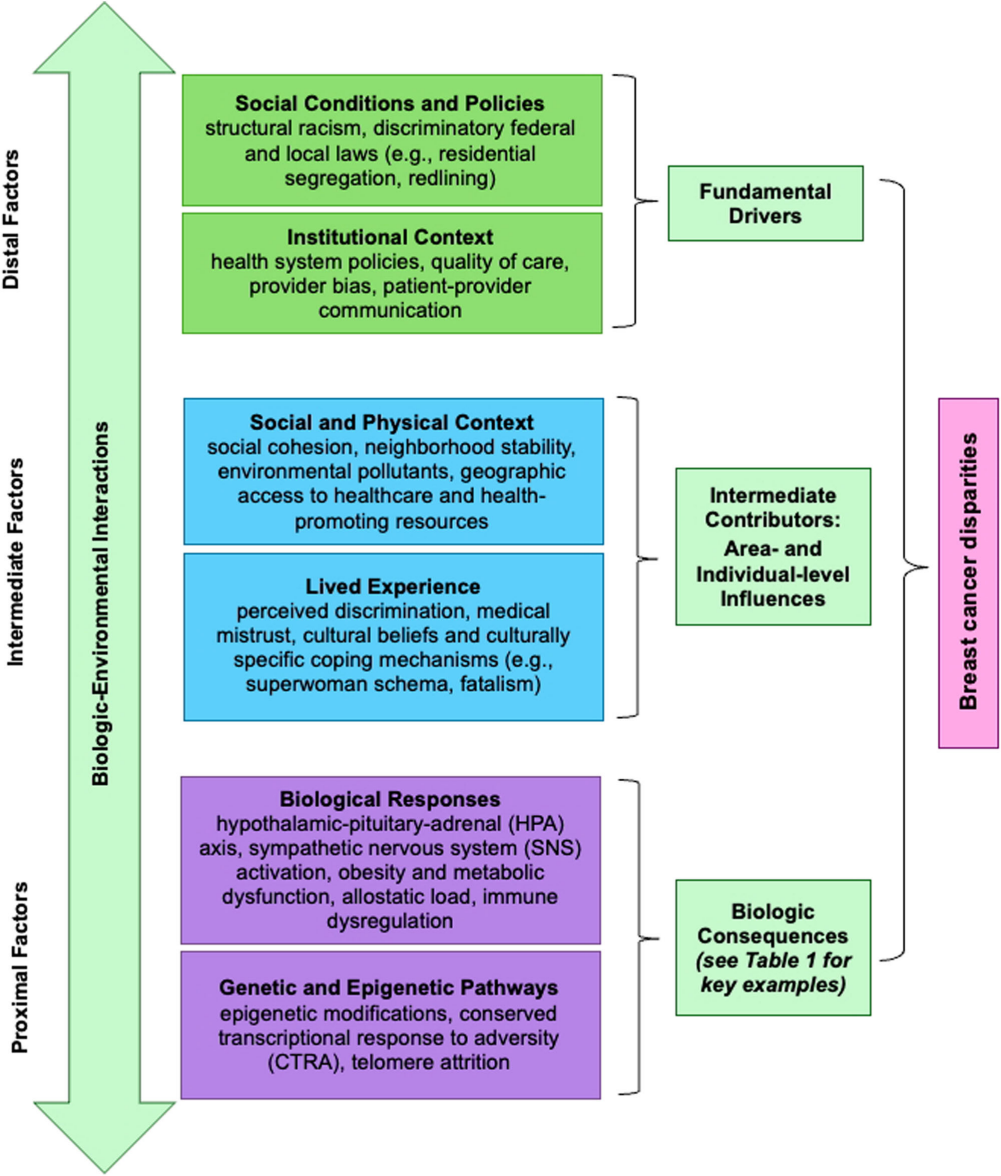
*Society-to-Cells* framework for understanding breast cancer disparities among Black women. Adapted from Warnecke’s Multilevel Model for Analysis of Population Health and Health Disparities^20^.

This review, guided by Warnecke’s Multilevel Model for Analysis of Health Disparities^20^, conceptualizes the determinants of breast cancer disparities across four interconnected levels: (1) fundamental drivers (e.g., structural racism, redlining, disinvestment), (2) area-level influences (e.g., social cohesion, neighborhood stability), (3) individual-level influences (e.g., lived experience, perceived discrimination, socioeconomic position, cultural beliefs)—which together represent intermediate contributors— and (4) biological consequences (e.g., allostatic load, stress physiology, genetic and epigenetic alterations, telomere attrition) (Fig. 1)^20^. This model underscores the interrelatedness of political, social, environmental, and biologic factors, grounded in the principle of downward causation—the idea that broad societal structures shape more immediate determinants of health^20^. For example, systemic disadvantage can influence neighborhood socioeconomic conditions, which in turn contribute to biologic stress responses that adversely affect health outcomes^21,22^. Framing this review through a *Society-to-Cells* lens, we explore how chronic exposure to structural and social adversity—rooted in systemic inequities and discriminatory policies, practices, and norms—become biologically embodied or “gets under the skin”^16,23,24^. These exposures influence cellular processes through mechanisms such as allostatic load, reflecting cumulative stress-induced wear and tear, and epigenetic modifications, both of which contribute to accelerated biological aging and, over time, increase susceptibility to adverse breast cancer outcomes^25–29^. The Warnecke model offers a comprehensive framework for understanding how multilevel drivers—from societal forces to cellular responses—interact to perpetuate cancer disparities. This approach provides a foundation for future efforts to reduce breast cancer disparities through multilevel interventions, including policy reform, investment in underserved communities, and dismantling systemic inequities that contribute to chronic stress in structurally marginalized populations. Importantly, the Warnecke model allows for reciprocal examination of factors across levels, rather than viewing them as strictly unidirectional^20^. While upstream structural drivers shape downstream outcomes, breast cancer and its treatment can produce downstream effects that, in turn, reinforce upstream drivers. For example, treatment-related financial hardship may lead to increased medical debt and risk of bankruptcy, constraining social mobility^30^. These financial hardships may also trigger residential relocation, which can disrupt social networks and continuity of care, thereby contributing to neighborhood disadvantage^31^ and greater exposure to adverse social and environmental determinants of health^32^. Thus, addressing this disparity not only demands attention to upstream drivers, but also the downstream consequences that perpetuate the cycle of inequity.

## Fundamental drivers

At the most distal level of the *Society-to-Cells* framework^20^, structural racism operates as a fundamental driver of breast cancer disparities, shaping the conditions in which Black women live, work, and seek healthcare. These conditions are influenced by political determinants of health, including discriminatory federal and local laws, zoning regulations, and patterns of systemic disinvestment^33^. One of the most well-documented examples is neighborhood redlining, a racially discriminatory federal housing policy established by the Home Owners’ Loan Corporation in the 1930s^34–36^. Through this policy, predominately Black neighborhoods were classified as “high-risk” for mortgage lending, reducing access to capital and triggering decades of disinvestment in education, healthcare infrastructure, housing, and economic opportunity^34–37^. The enduring effects of redlining illustrate how structural racism is not merely a historical artifact but an active, upstream determinant of health. These under-resourced environments expose residents to chronic social and environmental stressors—such as poverty, pollution, and community violence—while limiting access to high-quality healthcare, education, and employment^38^. Together, these forces generate the social and physical contexts that contribute to adverse health outcomes, including in breast cancer incidence, stage at diagnosis, and survival^16^.

### Redlining

Although formally outlawed by the Fair Housing Act of 1968, the legacy of redlining continues to influence breast cancer disparities, contributing to more aggressive tumor phenotypes, delayed diagnosis, and elevated mortality rates^19,39,40^. Black women residing in historically redlined neighborhoods have significantly higher odds of being diagnosed with aggressive breast cancer phenotypes, including a 62% higher odds of estrogen receptor (ER)-negative and 32% higher odds of triple-negative breast cancer^19^. Moreover, women living in these areas experience increased all-cause and breast cancer-specific mortality (hazard ratios [HR] 1.16 and 1.29, respectively) and are less likely to receive surgical treatment^40^. These patterns persist across generations: exposure to either historical redlining or contemporary mortgage discrimination is associated with a threefold increase in the risk of breast cancer mortality^19^.

Notably, the adverse effects of redlining are not fully mitigated by current neighborhood conditions. Individuals residing in historically redlined neighborhoods remain at elevated risk for late-stage diagnosis, regardless of present-day economic advantage^39^, highlighting the enduring consequences of structural disinvestment. Furthermore, the health impacts of redlining vary by race. NHW women benefit more from living in non-redlined neighborhoods, exhibiting lower odds of late-stage diagnosis, high tumor grade, triple-negative breast cancer, and breast cancer-specific death, compared to their Black counterparts^41^. These protective effects are not observed among Black women, suggesting that contemporary effects of redlining may perpetuate inequities by providing disproportionate benefits to privileged groups—likely through greater access to healthcare, improved socioeconomic conditions, and intergenerational transfer of wealth^41^. Together, these findings underscore how structural racism—manifested through redlining and sustained structural disinvestment—continues to shape the geographic distribution of risk, driving disparities in tumor biology, treatment access, and survival^19,38–41^. This evidence reinforces the importance of considering historical and contemporary structural drivers in understanding and addressing inequities in breast cancer outcomes.

### Residential segregation

Residential segregation, a persistent manifestation of structural racism, concentrates poverty, environmental hazards, and systemic disinvestment while limiting access to high-quality healthcare, education, employment, and other social resources^17,23,38^. These neighborhood-level disadvantages contribute to the perpetuation of socioeconomic inequities in predominantly Black communities and shape cancer risk and outcome across the life course. In a systematic review, residential segregation was associated with cancer disparities in 70% of included studies, including higher mortality and late-stage diagnosis^42^. These associations point to the mediating role of neighborhood environments, where factors such as limited access to healthy food, recreational space, and high-quality healthcare are common in segregated and under-resourced communities^20^.

Importantly, the health impacts of residential segregation persist even after adjusting for individual socioeconomic and insurance status, indicating that segregation is not merely a proxy for poverty, but a distinct structural determinant of health^42^. This pattern is echoed in breast cancer-specific studies, where residential segregation and neighborhood-level socioeconomic disadvantage are linked to both late-stage diagnosis and increased mortality, likely due to reduced access to screening, timely diagnosis, and high-quality treatment^43,44^.

Black women living in highly segregated metropolitan areas face the greatest risk, with HR for breast cancer mortality reaching 2.20 (95% CI: 1.09, 4.45)—a pattern not observed among White women, underscoring the racialized nature of structural exposure and unequal access to care^45^. Additionally, neighborhood physical disorder (e.g., visible signs of neglect such as litter, vandalism, and abandoned buildings) is more prevalent in racially segregated, and economically disadvantaged areas and associated with higher odds of late-stage breast cancer diagnosis and shorter survival, particularly among women diagnosed at an early stage^46–48^. Together, these findings highlight how structural racism, operationalized through residential segregation and neighborhood disinvestment, systematically concentrates social and environmental risks in marginalized communities while restricting access to health-promoting resources^45,46^. Within a *Society-to-Cells* framework^20^, these conditions set the stage for chronic stress exposure and adverse health trajectories that ultimately manifest in inequitable breast cancer outcomes. Importantly, the same structural forces that shape neighborhood disadvantage also permeate healthcare institutions, resulting in unequal access to high-quality care and differential treatment based on race—further compounding the effects of residential segregation.

### Healthcare system inequities

Structural racism not only shapes the environments where Black women live but also undermines health within the healthcare system itself, limiting access to high-quality care and perpetuating bias at the institutional and interpersonal levels^16,17,23^. Redlining and segregation-driven disinvestment in predominately Black communities have led to a scarcity of well-resourced healthcare facilities and a shortage of highly trained providers, restricting access to timely and high quality care^17^. These geographic inequities are compounded by provider-level biases—implicit and explicit—which contribute to differential treatment, reduced access to guideline-concordant care, and overall lower quality of care for Black individuals with cancer^41^.

Crucially, these disparities persist even after accounting for SES and insurance coverage, demonstrating that access and opportunity do not ensure equitable care^6,23,38^. As a result, Black women are simultaneously exposed to heightened health risks, while also receiving less effective treatment once inside the healthcare system. Across the breast cancer care continuum, Black women experience greater delays, including follow-up after abnormal mammograms, diagnostic biopsies, and initiation of treatment^11,49–52^. These delays are not fully explained by individual, neighborhood, or healthcare-level factors, suggesting deeper systemic barriers. In addition to delays, Black women are more likely to experience underuse or misuse of treatment and dose reductions due to treatment toxicity, which together contribute to later-stage diagnosis and poorer survival^8,11,12^.

Disparities also extend to symptom management and patient-provider communication. Black women report a greater symptom burden and more frequent undertreatment, even in clinical settings with standardized symptom assessments^53^. Inadequate symptom management can hinder treatment adherence and negatively impact survival^53^. Structural barriers such as lack of transportation, inflexible work schedules, and caregiving responsibilities further limit access to consistent, high-quality care^6,38,50^. Additionally, lower satisfaction with patient-provider communication and higher levels of medical mistrust are commonly reported among Black women with breast cancer^44,54^. These experiences are associated with psychological distress, including anxiety and depression, and may undermine treatment adherence^23,55^. Provider bias also plays a key role in shaping these interactions, influencing clinical decision-making and reinforcing systemic inequities^23,55^.

### Structural racism as the foundation of breast cancer disparities

Collectively, these findings illustrate how structural racism operates across multiple levels to drive breast cancer disparities—primarily through mechanisms such as redlining, residential segregation, chronic disinvestment, and systemic bias within the healthcare system. Structural racism increases exposure to health risks by concentrating socioeconomic disadvantage, degrading neighborhood infrastructure, and amplifying environmental hazards^17,55,56^. Within healthcare settings, it functions in more insidious and often unmeasured ways—such as through implicit provider bias and institutionalized discrimination—resulting in differential treatment and suboptimal care for Black patients^17,23,41,55^. Further, disenfranchisement and limited civic engagement (e.g., political underrepresentation, voter suppression) undermine the Black communities’ structural and political leverage to influence healthcare policy, demand equitable resource allocation, and hold healthcare institutions accountable^57^. Systemic barriers to political participation limit investment in essential healthcare services—such as prevention, screening, and treatment—and contribute to socioeconomic immobility, mistrust, and disengagement from healthcare systems^57^.

These interconnected pathways highlight that breast cancer inequities are not the result of individual behaviors or isolated risk factors, but rather are the product of structural forces embedded in policy, place, and institutional practice. As such, efforts to eliminate disparities must extend beyond clinical encounters to address the upstream political and structural determinants rooted in racism and systemic exclusion.

## Intermediate contributors: area-level influences

At the intermediate level of the *Society-to-Cells* framework, health is shaped by the social and physical contexts in which individuals live—contexts that are themselves deeply influenced by upstream structural drivers such as racism, segregation, and systemic disinvestment^20^. While these environments influence downstream behaviors and biological response, they are not random or neutral; they reflect historical and ongoing policies that determine the distribution of health-promoting and health-harming resources. Neighborhoods shaped by these structural forces often lack essential health-promoting infrastructure and expose residents to chronic social and physical stressors, which can suppress health-seeking behaviors and contribute to the biological embedding of disadvantage^17,23^. Consequently, health disparities must be understood not as the result of individual choices, but as the outcome of intersecting structural and contextual determinants.

In this review, we define the social context to include area-level factors such as social cohesion, neighborhood stability, and SES. The physical context includes environmental exposures and the availability of health-promoting resources—also at the area-level—such as access to healthcare, healthy food, safe spaces for physical activity, and opportunities for education and employment^27,58^. These examples are not exhaustive but serve to illustrate how upstream structural forces shape the broader context in which individual navigate their health. This level of the model aligns with the concept of deprivation amplification, in which individual vulnerabilities—such as low SES, perceived discrimination, and psychosocial stress—are intensified by adverse neighborhood conditions^58^.

Importantly, for Black women, the intersection of race and gender compounds these exposures leading to race- and sex-specific injustices^59^, including chronic discrimination, exposure to violence, financial strain^16,60,61^, and poverty^62^. These conditions heighten physiologic stress responses and reinforce disadvantage over time.

### Social and physical context

Social and physical contexts influence biological stress response pathways through mechanisms like allostatic load^63^, which, when chronically elevated, contribute to cancer progression and poor outcomes^16,64–68^. For example, neighborhood opportunity—a composite measure that includes transportation, education, employment, healthcare, housing, safety, and environment quality—is associated with both allostatic load and cancer survival outcomes^22^. Black Americans are disproportionately represented in lower-opportunity neighborhoods, which are associated nearly twice the allostatic load and a 45% increase in all-cause mortality, independent of individual SES^22^.

Neighborhood SES is another critical contextual factor. Although higher neighborhood SES is generally associated with improved 5-year survival across racial and ethnic groups, Black women consistently experience worse survival than White women at every SES levels^69^, even when quality of care is similar^11,12,14^. Strikingly, the mortality gap is greatest in high-SES neighborhoods, where Black women experience 64% higher mortality compared to White women, versus 21% in lower-SES neighborhoods^69^. Similarly, among those with health insurance, White women have higher cancer survival compared to Black women^70^. In contrast, there was no survival disparity comparing Black to White women among those who are uninsured. These findings highlight the potential for paradoxical relationships, suggesting that greater economic resources might lead to widening of racial disparities in breast cancer outcomes^69^.

The role of environmental pollutants in breast cancer disparities remains less well-defined. Some evidence finds no direct association between neighborhood-level pollution and breast cancer incidence among Black women^43^. However, the broader neighborhood stress environment—including crime, instability, and chronic psychosocial stressors—activate biological pathways and contributes to unhealthy coping behaviors^27,58^. Structural barriers such as limited access to healthy food and safe space for physical activity create an opportunity for risk of obesity and physical inactivity, which are known contributors to breast cancer progression and mortality^27,58^.

Within the social context, civic engagement shapes racial health disparities. Civic engagement through social cohesion (e.g., trust, belonging) and social capital (e.g., resources available through social connections), fosters resilience and empowers communities to engage in advocacy and political participation^57^. Black women living in predominantly Black, socially cohesive neighborhoods have improved breast cancer survival, potentially due to stronger social support networks that buffer stress, facilitate healthcare navigation, and reduced experiences of interpersonal discrimination^45,71^. In contrast, White women in these same neighborhoods may experience worse outcomes, possibly due to social isolation and weaker support systens^71^. These findings suggest that while racial residential segregation is often framed as detrimental to health, it may also promote community resilience and social capital among marginalized populations^16,45^. Highlighting the interplay between social contextual factors, one longitudinal study found that greater baseline interpersonal discrimination was associated with shorter telomere lengths 10 years later, but only among those reporting low neighborhood social cohesion^72^. Therefore, promotion of social cohesion and social capital in Black communities should consider participation in culturally appropriate religious spaces, social movements, and civic organizations that foster resilience, affirm racial identity, and build collective efficacy in the face of systemic oppression^57^. Collectively, these findings underscore the importance of equitable access to social and political determinants of health and equitable representation of Black women in policymaking, political leadership, and voting rights to create a system that supports and responds to their health needs^57^.

## Intermediate contributors: individual-level influences

Individual-level factors represent downstream manifestations of upstream structural and contextual forces. While public health efforts have traditionally focused on modifying individual behaviors—such as physical activity, diet, and screening participation—such approaches often overlook the structural conditions that constrain these behaviors. A shift in focus toward upstream social and structural determinants offers a more just and sustainable path to addressing breast cancer inequities. In this section, we examine how the lived experiences of Black women—including discrimination, coping responses, and culturally specific beliefs—interact with structural conditions to influence engagement with healthcare, psychological well-being, and biological stress responses that contribute to breast cancer disparities.

### Lived experience

Breast cancer disparities at the individual level are influenced by factors such as income, dietary patterns, cultural beliefs, and experiences of discrimination and medical mistrust. These factors affect how individuals engage with preventive care and treatment, and how they perceive and respond to environmental and structural stressors. Although resilience and culturally grounded coping strategies may offer some protection against adversity, these responses may be insufficient when compounded by persistent upstream stressors^73^.

Perceived discrimination is a chronic psychosocial stressor associated with adverse health across SES^56,74–76^. Discrimination contributes to chronic stress, elevated biomarkers of stress physiology, and increased breast cancer risk^16,23,77^. The biopsychosocial model developed by Clark et al. posits that the stress of perceived racisms contributes to poor health through sustained psychological and physiological wear^78^. Discrimination is also linked to emotional distress and health-compromising behaviors, and these pathways are increasingly understood to be biologically embedded^23^.

Culturally specific coping frameworks, such as the *Superwoman Schema*^79^, play a critical role in how Black women navigate stress. This schema is characterized by an obligation to project strength, suppress emotions, resist vulnerability, and prioritize caregiving. While these coping mechanisms can foster resilience, they are often developed in response to—or exacerbated by—social, political, and economic inequities^79^. Suppression of emotion and reluctance to seek help can intensify psychological distress and act as barriers to engaging in preventive care and treatment^80,81^.

Emerging evidence suggests that the *Superwoman Schema* also influences physiologic responses to discrimination. For instance, aspects such as emotional suppression and strength projection may buffer the effects of racial discrimination on stress physiology, while other components—such as intense obligations to care for others or succeed against the odds—may amplify biological stress responses, including elevated allostatic load^73^. These findings underscore the importance of considering culturally embedded coping mechanisms when examining the physiological effects of racism and their role in the biological embedding of health disparities^73^.

Fatalism, the belief that a breast cancer diagnosis inevitably results in death, is another culturally embedded belief that may influence screening behavior and treatment engagement^82^. Rooted in historical and community-level experiences rather than religious doctrine, fatalism can generate fear and denial, reinforce mistrust in medical systems, and serve as a barrier to timely engagement in preventive care^82^.

## Biologic consequences

Chronic and disproportionate exposure to stress across the life course, particularly among Black women, contributes to breast cancer disparities through physiologic dysregulation and biologic embedding^28,60,61,83^. These stress-related processes—rooted in structural racism and compounded by multilevel disadvantage and stress^38,55,78,84^—are associated with poorer health outcomes^16,61,64–67,85–87^, including increased breast cancer incidence^77^, and more aggressive tumor characteristics^88^.

Structural disadvantage is compounded by the lived experience of racial discrimination, a chronic psychosocial stressor^60^, that activates neuroendocrine pathways—notably the hypothalamic-pituitary-adrenal (HPA) axis and sympathetic nervous system (SNS)—which mediate downstream biological effects^17,24,56^. These intersecting social and biologic stressors (see Table 1) contribute to chronic immune dysregulation, including elevated proinflammatory cytokines (e.g., IL-6, TNF-$\alpha$) via activation of NF-kB and JAK-STAT3 signaling pathways^89,90^. These inflammatory responses promote tumor progression by stimulating angiogenesis, enhancing cell migration and invasion, and contributing to more aggressive phenotypes, such as triple negative breast cancer^91^. Furthermore, obesity-related inflammation and metabolic dysregulation—shaped by structural determinants—further exacerbates poor cancer outcomes^25,92–94^. Together, these processes are central to the *Society-to-Cells* framework, and represent the biologic consequences of upstream drivers^20^.

**Table 1:** Biologic pathways associated with psychosocial and structural stress in breast cancer: a Society-to-Cells perspective

Stress-Responsive Biologic Pathway	Messenger/Biomarker	Cellular Impact	Associations with Breast Cancer Disparities
Hypothalamic-pituitary-adrenal (HPA) axis activation/dysregulation (neuroendocrine)	• Cortisol • DHEA-S	• Neuroendocrine and immune dysregulation^28^ • Disrupted circadian rhythm^99,100^ • Altered glucocorticoid receptor sensitivity^67,112^ • DNA damage and p53 supression^98,101^ • Increased allostatic load (AL)^28,83^ • Promotion of angiogenesis, invasion, and migration via tumor microenvironment alterations^65^	• Chronic exposure to psychosocial and structural stressors, including racism, discrimination, and neighborhood disadvantage, activates the HPA axis and elevates cortisol in Black women^16,60,61^. This increases AL, particularly in the context of neighborhood level stressors^16,21,46,68^. • These changes accelerate biologic aging and disrupt immune surveillance and cell cycle control^67,99,100^. • May contribute to more aggressive tumor phenotypes and poorer clinical outcomes^16,64–67^.
Sympathetic nervous system (SNS) activation (neuroendocrine, Immune)	• Norepinephrine • Epinephrine	• $\beta$-adrenergic signaling (via cAMP/PKA, MAPK, PI3K)^103–105^ activates downstream NF-kB, AP-1, CREB, GATA transcription factors^89,104,105^ which increase pro- inflammatory cytokines (IL-6, TNF-$\alpha$, IL-1 $\beta$, IL-8)^127^ and decrease cytotoxic T cell activity^105^ • DNA damage and p53 supression^98,101^ • Promotion of angiogenesis, invasion, and migration^65,86,104,105^	• Chronic SNS activation promotes a pro-inflammatory, immunosuppressive tumor microenvironment^105^. • Elevated IL-6 and resistin levels in Black women promotes STAT3 activation and tumor aggressiveness, especially in triple negative breast cancer (TNBC)^91,94^. • SNS is an upstream driver of Conserved Transcriptional Response to Adversity (CTRA)^90^, which is associated with ER- breast cancer in Black women
Conserved Transcriptional Response to Adversity (CTRA) (transcriptomic, immune)	• IL-6, TNF- $\alpha$ • Type I interferon response genes	• Transcriptomic profile driven by $\beta$-adrenergic signaling leads to increased pro-inflammatory gene expression (IL-6, TNF- $\alpha$) and decreased antiviral defense^90^ • Suppressed Interferon regulatory factor (IRF) signaling impairs immune surveillance^90^	• CTRA reflects downstream transcriptomic response to chronic activation of stress pathways (SNS/HPA) in response to chronic exposure to psychosocial and structural adversity^89,90^. • Higher CTRA expression observed in Black women with ER- breast cancer, particularly in disadvantaged neighborhoods^122^ • Promotes inflammatory, immunosuppressed TME
Immune dysregulation	• IL-6 • TNF- $\alpha$ • C-reactive protein (CRP)	• Chronic inflammation, immune exhaustion, and decreased antiviral and antitumor immunity^127–130^ • JAK-STAT3 pathway activation leads to immune suppression and tumor growth^105^	• Chronic stress and obesity contribute to immune dysfunction and inflammation^25,26,93^ • IL-6 driven JAK-STAT3 signaling promotes tumor progression and impaired immune surveillance, particularly in Black women with TNBC^94,131^ • Reflects downstream consequences of HPA, SNS, and CTRA^90^
Metabolic dysregulation	• Insulin, • Leptin • Adiponectin • Reactive oxygen species (ROS)	• Insulin resistance and hyperinsulinemia • Activation of signaling pathways, including JAK-STAT3, PI3K-AKT, MAPK promote cell proliferation and survival^108^ • Oxidative stress • Increased aromatase activity leads to increased estrogen synthesis^93,107^	• Black women experience higher obesity rates due to structural inequities^16,17,92^. • Obesity promotes breast cancer, particularly ER+ and TNBC via inflammatory and hormonal pathways^93,106–108^ • Altered adipokines and inflammatory cytokines (e.g., leptin, IL-6) exacerbate tumor-promoting metabolism^93,94,131^.
Epigenetic modifications	• DNAm • Histone modification • microRNAs	• Altered methylation of genes (e.g., NR3C1, WWOX)^110–114,132^ • Modulates immune signaling, stress responsivity, and oncogenic pathways (e.g., tumor suppressor genes) • Contributes to cellular aging	• Early life adversity associated with altered DNAm patterns^16^, including NR3C1 DNAm and impaired HPA axis regulation^112,133^ • Perceived discrimination associated with DNAm of tumor suppressor gene, *WWOX*^113,114^.
Telomere biology	• Shortened relative telomere length (RTL)^29,116^	• Genomic instability • Impaired DNA repair • Cellular senescence and accelerated biological aging	• RTL shortening and accelerated aging more pronounced in Black women (~7.5 years)^72^ and associated with chronic stress and discrimination^16,27,95,115–117^ with greater RTL attrition in neighborhoods with low social cohesion^72^. • Premature RTL shortening associated with increased cancer risk, recurrence, and all-cause mortality^118–120^
Allostatic load (multisystem dysregulation)	• Composite indices incorporating numerous markers from cardiovascular, metabolic, immune, and neuroendocrine systems	• Wear and tear across neuroendocrine, metabolic, immune and cardiovascular systems^63,97^ • Mitochondrial dysfunction	• Allostatic load elevated among Black women^16,21,25,28^ • Higher allostatic load associated with more aggressive breast tumor phenotypes, poorer quality of life, and poorer clinical outcomes^68,121,134^ • Reflects cumulative impact of structural and psychosocial stressors^83^

### Biological response: select examples

Biologic embedding refers to the process through which social and environmental stressors become internalized at the molecular and cellular level, manifesting in alterations such as DNA methylation and telomere attrition^16,28,95,96^. Allostatic load, by contrast, represents the cumulative “wear and tear” on the body’s systems—including the cardiovascular, metabolic, immune, and neuroendocrine—as they adapt to chronic stress^28,63,83,97^.

While evidence supports the influence of social and structural determinants on breast cancer outcomes, the precise mechanisms linking context to tumor biology remain under investigation. For example, neighborhood-level exposures—historically shaped by structural racism—can disrupt allostasis and drive multisystem dysregulation^21,22^. Chronic activation of the HPA axis disrupts circadian cortisol rhythms, alters tumor cell proliferation, and promotes DNA damage^98–100^. Elevated cortisol reduces tumor suppressor proteins such as p53^98,101^, and remodels the breast tumor microenvironment (TME) to facilitate tumor angiogenesis, cell migration, and immune invasion^65,67,86,89^. In parallel, SNS-mediated β-adrenergic signaling further exacerbates DNA damage and upregulates pro-inflammatory gene expression by activating transcription factors such as NF-kB, AP-1, and GATA. This signaling can also suppress p53 activity^98,101^, impairing DNA repair and promoting tumor progression^65,67,86,89,102–105^.

Activation of the HPA axis and SNS also drives metabolic dysfunction, including insulin resistance and dysregulated adipokine signaling, contributing to a TME marked by elevated insulin, glucose, and inflammatory cytokines^93,106^. Adipose tissue increases estrogen production through aromatase expression, fueling ER-positive tumor growth^25,93,107^. Adiposity induced hyperinsulinemia, inflammation and hormonal imbalances activate pro-tumorigenic signaling pathways such as JAK-STAT3, PI3-AKT and MAPK, further promoting cellular proliferation and angiogenesis^106,108^. Adipokines (e.g., leptin) and inflammatory cytokines (e.g., TNF-$\alpha$, IL-6) further contribute to this tumor-promoting microenvironment^93,106^.

### Genetic and epigenetic pathways: select examples

Epigenetic modifications, such as DNA methylation (DNAm), contribute to the biologic embedding of chronic stress by silencing tumor suppressor genes and activating oncogenes^109–111^. For instance, DNAm of the *NR3C1* gene, which encodes the glucocorticoid receptor, has been linked to early-life adversity and observed in breast tumor tissues^16,112^. These epigenetic alterations may disrupt cortisol regulation and increase HPA axis reactivity in breast epithelium^16,112^. Similarly, perceived discrimination has been associated with DNAm of *WWOX*^113^, a tumor suppressor gene implicated in breast cancer pathogenesis^114^.

Relative telomere length is another key marker of physiologic stress^28,95,115–117^. Telomeres protect chromosomal integrity, and their premature shortening is associated with accelerated biological aging, increased risk of breast cancer recurrence, and all-cause mortality^28,118–120^. Notably, Black women show greater telomere attrition, corresponding to an accelerated aging equivalent of ~7.5 years compared to White women^29^.

Allostatic load is also associated with increased leukocyte mitochondrial DNA copy number, suggesting a role for mitochondrial dysfunction in the stress-related biologic embedding^26^. Among Black women, elevated allostatic load has been linked to more aggressive tumor phenotypes, as well as poorer quality of life and clinical outcomes^25,26,68,121^. These findings reinforce the need to examine sociobiologic mechanisms—including life course stress exposure, contextual adversity, and structural inequality—as critical contributors to racial disparities in breast cancer^16,61,78^.

Relatedly, the Conserved Transcriptional Response to Adversity (CTRA) is a stress-related gene expression pattern^89,90^ characterized by the upregulation of pro-inflammatory genes (e.g., IL-6, TNF-$\alpha$) and the downregulation of genes involved in antiviral and antibody-related responses, such as type I interferon pathways^89,90^. Chronic activation of the SNS and β-adrenergic signaling pathways drives CTRA, which modulates immune cell gene expression through transcription factors such as NF-kB and AP-1, along with decreased activity of interferon regulatory factors (IRFs)^104,105^. These transcriptional shifts contribute to chronic inflammation, suppressed antiviral defenses, and impaired cytotoxic immune function^89,90,104,105^.

CTRA-driven immune shifts promote a pro-inflammatory TME, which may be especially relevant for ER- negative breast cancers^122^. Thus, the CTRA exemplifies biologic embedding within the *Society-to-Cells* framework, wherein chronic exposure to structural and psychosocial stressors and adversity drives molecular and immunologic changes that impact disease^89,90^.

While our framework conceptualizes race as a social construct shaped by structural and social determinants, emerging evidence suggests that West African genetic ancestry may be associated with differential risk for certain breast cancer subtypes^123–126^. While population-level genomic variation may contribute to breast cancer risk, exploring ancestry-related genetic factors is beyond the scope of this review and not central to the *Society-to-Cells* framework guiding our analysis^20^.

## Conclusion—from Society to Cells: implications for research and equity

In conclusion, this review underscores how structural racism—manifested through policies such as redlining, patterns of residential segregation, and systemic disinvestment—fundamentally shapes health across multiple levels. These upstream forces influence the social and physical environments in which individuals reside, access healthcare, and experience daily discrimination and stress. Cumulative exposure to these multilevel disadvantages drives biological responses, including elevated allostatic load and biological embedding, which are associated with increased breast cancer incidence^77^, more aggressive tumor phenotypes^88^ and poorer clinical outcomes^16,64–68^. This *Society-to-Cells* perspective makes visible how systemic inequities are literally integrated into the body.

Guided by Warnecke’s Multilevel Model for Analysis of Health Disparities^20^, this review illustrates the interconnected pathways by which structural racism, contextual disadvantage, and lived experience become biologically embedded, ultimately contributing to persistent racial disparities in breast cancer outcomes. Addressing these disparities demands more than individual-level behavior change—it requires systemic reforms, including structural policy interventions, reinvestment in historically marginalized neighborhoods, and the elimination of bias within healthcare systems.

As it stands, the system not only exposes Black women to greater cumulative risk—via environmental stressors, inadequate infrastructure, and restricted access to health-promoting resources—but also delivers lower-quality care when accessed. These inequities are not isolated or incidental – they are structurally produced and biologically consequential.

## Data Availability

No datasets were generated or analysed during the current study.
